# Comparison of a video versus paper questionnaire on functional limitation in lower limb osteoarthritis

**DOI:** 10.1186/s12891-019-2868-6

**Published:** 2019-11-03

**Authors:** L. Dubouis, W. Ngueyon-Sime, W. Peter, A. Vallata, J. Epstein, A-C. Rat, N. Agrinier, C. B. Terwee, F. Guillemin

**Affiliations:** 1 CHRU-Nancy, Inserm, Université de Lorraine CIC Clinical epidemiology, Nancy, France; 2 Amsterdam UMC, Vrije Universiteit Amsterdam, Department of Epidemiology and Biostatistics, Amsterdam Public Health Research Institute, de Boelelaan 1117, Amsterdam, the Netherlands; 3 Université de Lorraine, APEMAC, Nancy, France

**Keywords:** Questionnaire, Osteoarthritis, Function, Video

## Abstract

**Introduction:**

The video Animated Activity Questionnaire (AAQ) was developed to assess the impact of lower limb osteoarthritis (OA) on daily functional activities. The objective of the study was to compare the video and the HOOS/KOOS paper questionnaires and to assess the effect of order of administration.

**Material and methods:**

Patients recruited in the KHOALA cohort were randomized in two groups: AAQ questionnaire first (AAQ-first group) and HOOS (hip)/KOOS (knee) questionnaire first (H/KOOS-first group). Within group differences between AAQ and HOOS/KOOS scores were compared using a Student t-test. The Spearman correlation coefficient between AAQ score and HOOS/KOOS score was calculated in each group then compared, using Fisher z-transformation.

**Results:**

Among 200 randomized patients, 188 (65.8 years, 66.0% women) completed the questionnaires: 99 in the AAQ-first group and 89 in the H/KOOS-first group. The AAQ score was 85.9 (SD: 13.7) in the AAQ-first versus 87.8 (SD: 13.1) in the H/KOOS-first group (*p* = 0.34). The H/KOOS score was 72.5 (SD: 21.2) in the AAQ-first versus 73.5 (SD: 18.4) in the H/KOOS-first group (*p* = 0.71). The Spearman correlation coefficient between AAQ and H/KOOS in the AAQ-first was 0.84[0.77–0.89] and 0.73[0.61–0.81] in H/KOOS-first group. These correlations differed between groups significantly (*p* = 0.02).

**Conclusion:**

This study found video AAQ and paper HOOS/KOOS questionnaire highly correlated, with a moderate but significant effect of order administration of video and paper questionnaires evidencing a stronger correlation when the videos were viewed first.

## Introduction

Osteoarthritis (OA) is the most frequent joint disease. The hip and knee OA, the most disabling, account for 9,1% of women (about 3 million women) and 6,6% of men (about 2 million men) among 40–75 years old in France [1]. OA progresses by flare followed by chronic pain with an impact on daily activity and function. Several methods are available to assess this impact. For instance, performance-based tests [2, 3], where a health professional observes OA impact on patients daily activities. They are difficult to conduct on a regular basis, requiring staff personal, and are costly. Patient Reported Outcome Measures (PROMs) [4] have been developed to allow patient self-evaluation. In OA the Hip disability Osteoarthritis Outcome Score (HOOS) [5] and the Knee injury Osteoarthritis Score (KOOS) [6] are commonly used. The degree of severity expressed is then depending on the representation the patient has of her/his disease [7].

In order to correctly assess the impact of OA on daily basic functional activities such as walking, rising and sitting down, and stair climbing, an interactive questionnaire has been developed in close collaboration with two focus group of hip and knee OA patients [8]. The Animated Activity Questionnaire (AAQ) [8, 9] is a computerized video questionnaire that assesses daily basic activity limitations. The psychometric properties of the AAQ have been established in an international study [8, 9] demonstrating its reproducibility and expected correlations between paper and video questionnaires, administered in this order, and scale calibration thanks to differential item functioning (DIF) analysis [10].

One may hypothesize that the video questionnaire, when administered first, influences the responses of the patients to paper questions on their abilities and functional capacities. Randomizing the order of administration will allow detecting such an effect and, in contrast, asserting independent measurement of each questionnaire if none is evidenced.

The objective of this study was to assess the effect of order of administration of video AAQ and paper HOOS/KOOS questionnaires on responses.

## Patients and methods

### Sampling

This study was an ancillary analysis of an international validation study of the AAQ questionnaire [10] in which we introduced a randomization of order of administration in the French sample, conducted from March 2014 to June 2015.

Patients were recruited in the KHOALA cohort [11], aged 40 to 75 years at inclusion in the cohort, and presenting knee or hip OA diagnosed and confirmed by a rheumatologist according to American College of Rheumatology (ACR) Clinical Criteria [12, 13]. Exclusion criteria were: unable to fill in questionnaires, no informed consent. Furthermore to be included in the present study, they had to receive some treatment for osteoarthritis in the past 12 months.

The sample size was imposed on by the international multi-country protocol. Among 200 randomized patients, 189 patients were eligible to complete the questionnaires in the 2 groups (90 in HOOS/KOOS-first and 99 in AAQ-first groups). One patient from the HOOS/KOOS-first group with missing data was excluded.

### Ethical approval

This study was approved by the Medical Ethics Committee of France (CPP Est III Nancy) and institutional national review board (CNIL*)*. The study complied with the Declaration of Helsinki. Patients provided written informed consent.

### Randomization

Patients satisfying inclusion criteria were computer randomized at the Inserm CIC coordinating center in two arms; AAQ questionnaire first followed by H/KOOS questionnaire (AAQ-first group) or H/KOOS questionnaire first followed by AAQ questionnaire (H/KOOS-first group).

### Data

Data were collected before a clinic visit, alone in the room. They included socio-economic characteristics: age, sex, BMI; clinical characteristics: symptomatic joint (hip, knee or both), side (right, left or both) and pain (VAS); and PROMs questionnaires without missing data (HOOS, KOOS, and AAQ).

The score of HOOS and KOOS questionnaires, referring to the more symptomatic joint, contains 17 items each scored 0 to 4. The full score obtained by the sum of items was standardized to 0–100 points scale with higher score representing less limitations in daily activity.

The AAQ video questionnaire is composed of 17 videos showing daily life situations: going up and downstairs (2 items); walking outside on a flat surface (1 item); walking outside on uneven terrain (1 item); walking inside (1 item); walking up or down a slope (2 items); picking up an object from floor (1 item); rising and sitting down on the floor (1 item); rising and sitting on the chair, sofa, and toilet (6 items); putting on and taking off shoes (2 items). For each activity, three to five videos show to patient the levels of difficulty. In addition a response option ‘unable to perform the activity’ is given. For each activity all videos are shown on the same screen to facilitate comparison from the first with optimal performance to the last with the maximum level of disability. Videos are visualized as many times as needed. The patient must chose the video that best corresponds to how he/she is performing that activity. A research nurse was available for guidance and checked patients filled in the questionnaire until the end item.

Each activity is scored from 0 to 4, 5 or 6 according to the number of videos, and the option ‘unable to perform’. Each activity score is translated on a 0 to 100 scale. The total AAQ score is the sum of all activities divided by 17. The higher score on the 0 to 100 scale corresponds to less activity limitations.

The HOOS, KOOS and AAQ questionnaires have shown good validity, internal consistency, test-retest reliability in source and French languages [5, 6, 8–10].

### Statistics

Patients baseline characteristics were described per group. The characteristics of these 2 groups were compared using the Chi-square test for qualitative variables. The quantitative variables, and the within group difference between the AAQ and HOS/KOOS scores were compared Student t test.

The Spearman correlation coefficient between AAQ score and HOOS/KOOS score was calculated in each of the 2 groups to assess the intra-group correlation. These coefficients were then compared after Fisher z-transformation by a Student t test.

Since the sample size was constrained by the international study protocol, a post-hoc power was calculated.

Statistical analysis was done using SAS Version 9.3, Cary NC, USA.

## Results

**Fig. 1:**
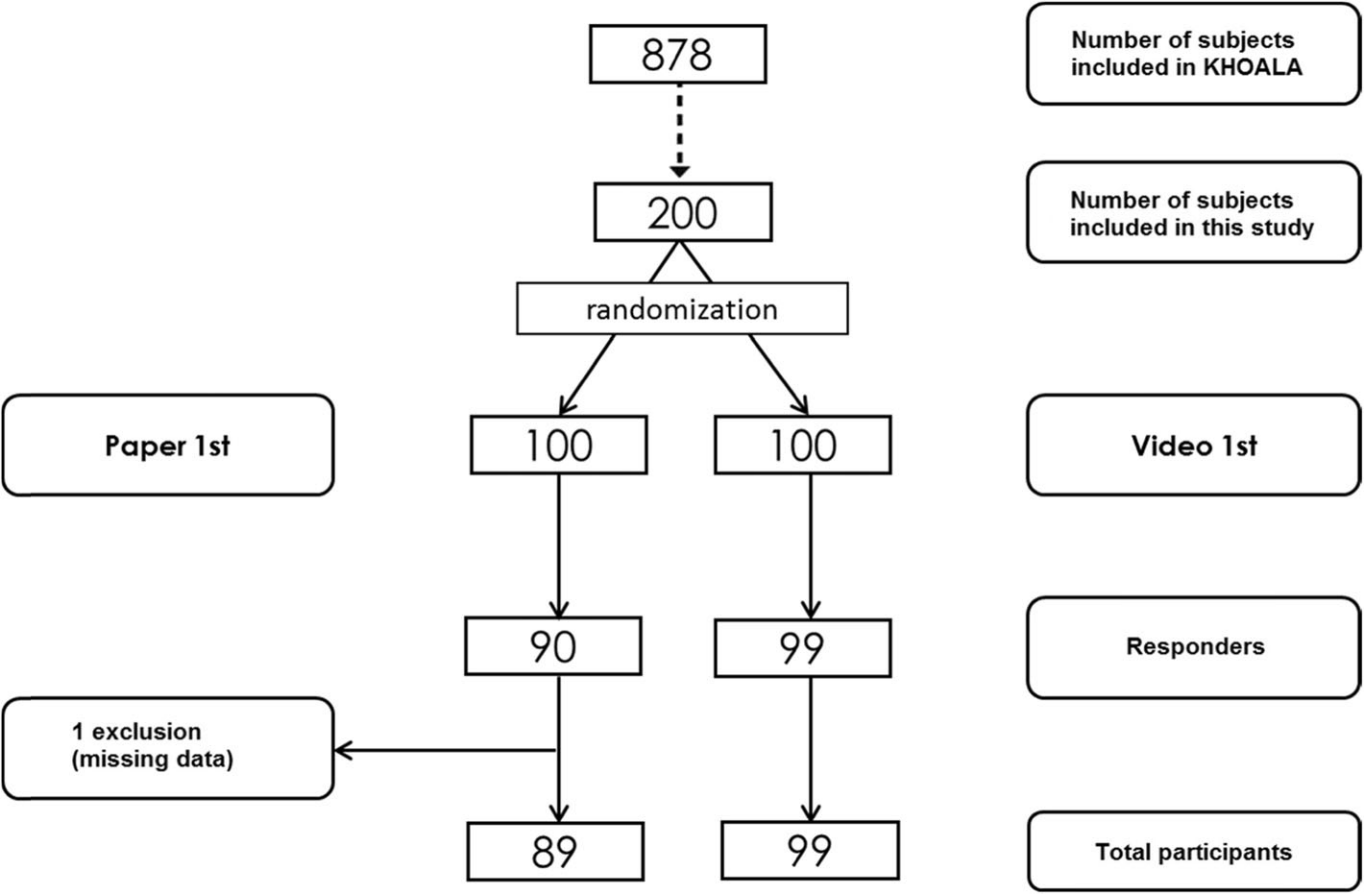
Patient’s selection from the KHOALA cohort

A total of 188 patients with complete data were included (Fig. 1). Mean age was 65.8 years with 2:1 women to men ratio. The patients who answered the questionnaire were significantly older than those who did not (65.8 vs 58.9 years; *p* < 0.0001) and showed no difference in sex, level of education and BMI. The mean AAQ score was 86.8 (SD: 13.4). The mean HOOS/KOOS was 73.0 (SD: 19.9) and the mean difference between AAQ and HOOS/KOOS was 13.8 (SD: 11.9).

Patients characteristics in each randomized group showed no difference (Table 1). Neither the AAQ scores in the AAQ-first group versus in the HOOS/KOOS-first group (*p* = 0.34)), nor the HOOS/KOOS scores in the AAQ-first group versus in the HOOS/KOOS-first group (*p* = 0.71)) differed.

**Table 1:** Comparison of groups according to questionnaires order of administration (*n* = 188)

	AAQ-first group			HOOS/KOOS-first group			
	*N* = 99 (52.7%)			*N* = 89 (47.3%)			
	N	%/mean	SD^a^	N	%/mean	SD^a^	p^b^
Age	99	65.9	7.5	89	65.6	6.9	0.75
Sex							0.69
Male	35	35.4		29	32.6		
Female	64	64.6		60	67.4		
Body Mass Index	97	28.4	5.3	88	28.6	6.6	0.82
Level of education							0.39
Primary	12	12.1		17	19.1		
Secondary	51	51.5		40	44.9		
Superior	36	36.4		32	36.0		
Pain	99	3.5	2.3	89	3.4	2.3	0.86
Site							0.56
Hip	28	28.3		21	23.6		
Knee	60	60.6		54	60.7		
Both	11	11.1		14	15.7		
Side							0.95
Right	39	39.4		35	39.3		
Left	36	36.4		34	38.2		
Both	24	24.2		20	22.5		
AAQ score	99	85.9	13.7	89	87.8	13.1	0.34
HOOS/KOOS score	99	72.5	21.2	89	73.5	18.4	0.71

^a^ *SD* Standard deviation

^b^ Chi-Square test for qualitative variables and Student t test for quantitative variables

The Spearman correlation coefficient between AAQ and HOOS/KOOS questionnaires was 0.84 [0.77–0.89] in the AAQ-first and 0.73 [0.61–0.81] in the H/KOOS-first group and these coefficients differed significantly (*p* = 0.02). These results did not differ by hip or knee joint (data not shown). The power of this analysis was 93.3%.

## Discussion

This study found a significant effect of order administration of video AAQ and paper HOOS/KOOS questionnaires. The AAQ and HOOS/KOOS scores were more highly correlated in patients who completed the AAQ first, indicating that seeing the video images first may have a stronger impact on answering paper questionnaire than the reverse in the domain of physical mobility. The absence of difference in correlations would have suggested that the two questionnaires, measuring different though correlated constructs, influence each other equally.

To be consistent, patients may adapt their responses to the second questionnaire depending on responses given to the first, i.e. a halo effect [14], modifying their perception to evaluate specific aspects of functional limitation contained in the second questionnaire. When a patient completes several questionnaires (with the same answer scale but on a different administration mode), the subject tends unconsciously to modify his answers to the second questionnaire so that they correspond to the first and thus be faithful to itself. Moreover, it is likely that showing the video representation first imprints responses to the paper second while reciprocal is less true assuming that videos has more impact than words [15].

In this experiment, the absolute difference in scores between the two questionnaires cannot be interpreted, since the exact correspondence in content between the two constructs is not the same, and neither are the metrics used.

The AAQ questionnaire combines advantages of PROMs and of performance-based tests without most of their inconveniences: decrease of inter-subjects variability for interpretation, independence of patient motivation to do the test, videos referring to activities in the past week and not to a single time of the test performance. The cost is low and the comprehension is easier as it is not text dependent. However, its impact on the responses to subsequent questionnaires should be kept in mind.

This study has some limitations. Although the patients were sampled in a national cohort representative of French knee and hip osteoarthritis, it was only one country and the results may not be fully extrapolated to other. However, the international AAQ validity study [9] has shown the measurement robustness of the AAQ instrument. Therefore, it is plausible that these results can be generalized. Second, the absence of a gold standard (like an external observer quoting the patients performances) does not allow us to infer whether one measure is less biased than the other. Another strengths of this study is its statistical power (93.3%).

## Conclusion

To conclude, the AAQ and HOOS/KOOS scores were more highly correlated in patients who completed the AAQ first than in patients who completed the HOOS/KOOS first, indicating a stronger association when videos are viewed first.

## Data Availability

The datasets used and analysed during the current study are available from the corresponding author on reasonable request.
